# Isolate Dependency of *Brassica rapa* Resistance QTLs to *Botrytis cinerea*

**DOI:** 10.3389/fpls.2016.00161

**Published:** 2016-02-17

**Authors:** Wei Zhang, Soon-Tae Kwon, Fang Chen, Daniel J. Kliebenstein

**Affiliations:** ^1^Department of Plant Sciences, University of California, Davis, DavisCA, USA; ^2^National and Local Joint Engineering Laboratory for Energy Plant Bio-oil Production and Application, Key Laboratory of Bio-resource and Eco-environment, Ministry of Education, College of Life Sciences, Sichuan UniversityChengdu, China; ^3^Department of Horticulture and Breeding, Andong National UniversityAndong, South Korea; ^4^DynaMo Center of Excellence, University of CopenhagenCopenhagen, Denmark

**Keywords:** plant–pathogen interaction, *Brassica rapa*, *B. cinerea*, quantitative disease resistance, QTL mapping, GSL

## Abstract

Generalist necrotrophic pathogens including *Botrytis cinerea* cause significant yield and financial losses on *Brassica* crops. However, there is little knowledge about the mechanisms underlying the complex interactions encoded by both host and pathogen genomes in this interaction. This potentially includes multiple layers of plant defense and pathogen virulence mechanisms that could complicate in breeding broad spectrum resistance within *Brassica* species. Glucosinolates (GSLs) are a diverse group of defense metabolites that play a key role in interaction between *Brassica* and biotic attackers. In this study, we utilized a collection of diverse *B. cinerea* isolates to investigate resistance within the *Brassica rapa* R500 × IMB211 recombinant inbred line population. We tested variation on lesion development and glucosinolate accumulation in parental lines and all population lines. We then mapped quantitative trait loci (QTL) for both resistances to *B. cinerea* and defense metabolites in this population. Phenotypic analysis and QTL mapping demonstrate that the genetic basis of resistance to *B. cinerea* in *B. rapa* is isolate specific and polygenic with transgressive segregation that both parents contribute resistance alleles. QTLs controlling defensive GSLs are highly dependent on pathogen infection. An overlap of two QTLs identified between resistance to *B. cinerea* and defense metabolites also showed isolate specific effects. This work suggests that directly searching for resistance loci may not be the best approach at improving resistance in *B. rapa* to necrotrophic pathogen.

## Introduction

Generalist necrotrophic pathogens including *Botrytis cinerea* are significant economic challenges on *Brassica* crops because of its ability to cause lesions on nearly all harvestable components of the plant including leaves, stems, florets, and pods (Bolton et al., 2006; Williamson et al., 2007). Thus, there is interest in breeding for resistance to *B. cinerea* within *Brassica* crop species. This is, however, complicated because resistance to *B. cinerea* is a highly quantitative trait with little evidence of major effect resistance loci (Denby et al., 2004; Finkers et al., 2007a,b, 2008; Rowe and Kliebenstein, 2008; Staal et al., 2008; Davis et al., 2009; Anuradha et al., 2011). The effort if further complicated by the desire to breed for broad spectrum resistance which runs into the complication that *B. cinerea* is a highly polymorphic pathogen with this genetic variation altering the virulence mechanisms by which the pathogen interacts with the plant (ten Have et al., 1998; Denby et al., 2004; Rowe and Kliebenstein, 2007, 2008; Amselem et al., 2011; Staats and van Kan, 2012). However, most genetic or molecular studies looking at how plants resist *B. cinerea* typically utilize individual isolates hindering the effort to find loci that provide potential resistance to a broad spectrum of *B. cinerea* isolates.

Defense metabolites including glucosinolates (GSLs), have frequently been linked to resistance to *B. cinerea* and other generalist necrotrophic pathogens within the *Brassicas* (Stotz et al., 2011; Buxdorf et al., 2013; Cargnel et al., 2014; Calmes et al., 2015). GSLs are sulfur containing secondary metabolites unique to the order Capparales whose genetics and chemistry have been extensively studied (Chan et al., 2010; Sønderby et al., 2010; Kliebenstein, 2014). In addition to necrotrophic resistance, these compounds also alter resistance to biotrophic pathogens, insects and aphids indicating that they are likely key players in numerous biotic interactions of *Brassica* plants (Kroymann and Mitchell-Olds, 2005; Pfalz et al., 2007, 2009; Fan et al., 2011; Weis et al., 2014; Kerwin et al., 2015). As a result of this role in numerous different biotic interactions, GSLs show extensive genetic variation in all tested *Brassica’s* but the link between this natural genetic variation and quantitative resistance to pathogens including *B. cinerea* has not been queried (Kliebenstein et al., 2002a,b; Wentzell et al., 2007; Chan et al., 2010; Velasco et al., 2011; Feng et al., 2012; Zou et al., 2013; Rahman et al., 2014; Brown et al., 2015; Gupta et al., 2015; Rout et al., 2015).

To test the influence of genetic variation in *B. cinerea* upon quantitative resistance in *Brassica rapa*, we utilized a collection of 14 genetically variable *B. cinerea* isolates to test for variation in lesion development on the IMB211 and R500 parents of a *B. rapa* RIL population (Iniguez-Luy et al., 2009). This identified a significant interaction of the host and pathogen genotypes on the quantitative resistance outcome of the interaction. We then proceeded to map resistance to five *B. cinerea* isolates in the IMB211 × R500 RIL population. Simultaneously, we measured glucosinolate accumulation in all the RILs in the presence and absence of the pathogen to map defense metabolite quantitative trait loci (QTLs). This showed that the detached leaf assay approach identified isolate specific resistance QTLs in *B. rapa* and that the defense metabolite QTLs were highly dependent upon the interaction with the pathogen. There was also an overlap of two QTLs between defense metabolites and resistance to *B. cinerea* but both loci showed isolate specific effects. This work suggests that a potential avenue going forward to breed for quantitative resistance to *B. cinerea* in *B. rapa* would be to focus on breeding for the proper defense metabolite blend. Or alternatively to stack isolate specific resistance loci to create the appearance of broad spectrum resistance. Further work is required to assess if these detached leaf identified loci will work in whole plant field based assays.

## Materials and Methods

### Bulking of *Brassica rapa* Germplasm

Seeds of the two parental lines of the *B. rapa* BraIRRi population, the annual yellow sarson R500 (male) and the rapid cycling IMB211 (female) (Williams and Hill, 1986) as well as the resulting recombinant inbred lines (RILs) population, were obtained (Iniguez-Luy et al., 2009). Both parental lines and 133 lines of RILs in this study were bulked during 2013 in the University of California, Davis greenhouses under a controlled environment. The plants were grown under a 12-h photoperiod under metal-halide lamps using a day/night temperature set at 25/18°C and relative humidity of 70. Plants were grown in 15-cm-diameter plastic round pot, filled with standard potting soil (Sunshine Mix #1; Sun Gro Horticulture) and ∼4.5 g of a slow-release fertilizer (14-14-14 Osmocote, Scotts). For bulking, all plants were bagged with mesh pollination bag during flowering to prevent cross-pollination. The plants were staked upright to produce larger fruits and reduce pathogen and herbivore attack. We watered the plants once a day and then reduced the watering times for about 2 weeks when the fruits began to mature. The seeds were harvested once the majority of the seedpods were dry and then stored separately in paper bags in a cool, dry, dark place until further use.

### Growth of the R500 X IMB211 RIL Mapping Populations for QTL Analysis

For measuring the resistance to *B. cinerea* and GSL metabolite accumulation, the *B. rapa* parental lines and 120 RILs that had sufficient seed were raised in a controlled environment chamber at University of California, Davis. Three seeds of each genotype were sown in the center of a separate well of a 6 × 12 well tray filled with standard potting soil matrix and the tray was placed in a large planting flat (280 mm × 540 mm × 58 mm). All genotypes were randomized in a randomized complete block design. Two liters of nutrient-enriched water (0.5% N-P-K fertilizer in a 2-1-2 ratio; Grow More 4-18-38) were added into the flat to ensure the compost around seeds was moist. The flat was covered with a transparent plastic hood to maintain humidity during germination and placed into a chamber at 5°C to complete vernalization. After 3 days of chilling, the transparent hood was removed and the flat was transferred into a climate-controlled chamber with the temperature at 22°C and a photoperiod 10 h light: 14 h dark photoperiod. All plants were watered twice a week using nutrient-enriched water. At 21 days after sowing, 4–5 true leaves were harvested from each plant for analysis of lesion size and GSL content. The entire experiment was repeated a second independent time.

### *B. cinerea* Isolates, Preparation of Conidia and Inoculation

Information for all *B. cinerea* isolates used in this study is described in previous reports (Denby et al., 2004; Kliebenstein et al., 2005). To collect spores for plant innoculation, all isolates were maintained as conidial suspensions in 30% glycerol at -80°C at our lab for long time storage. Conidia suspensions were swabbed on freshly prepared potato dextrose agar (PDA, Gibco/Invitrogen, Carlsbad, CA, USA) medium in Petri dishes and cultured at room temperature. Spores used for infection on *B. rapa* leaves were obtained as described (Rowe and Kliebenstein, 2008). The detached leaf assay has been utilized in numerous settings to identify causal loci controlling resistance to necrotrophic fungi. While this assay will miss loci controlling resistance in a whole plant context like pedicel transmission barriers, it is a useful approximation (Sharma et al., 2005; Mulema and Denby, 2012; Cowley et al., 2014; Boydom, 2015). For lesion assays and trypan blue staining, the fully developed detached leaves placed on 1% phytoagar in large plastic trays. Detached leaves were inoculated with 4 μL droplets of *B. cinerea* spore suspensions (10 spores/uL) in 50% filtered grape juice (Santa Cruz Organics, CA) at room temperature with light illumination. Control leaves (mock) were inoculated with of the 4 μL droplet of 50% filtered grape juice without spores. An abiotic GSL elicitor, AgNO3 (5 mM), was inoculated in the same way. Six independent infections were conducted per isolate/genotype pair across the two independent experiments. Digital photographs were taken every 8–12 h to examine the lesion development on leaves.

To test for differences in the *B. rapa* parental lines, we screened fourteen *B. cinerea* isolates for differential virulence against the two parental lines using six independent biological replicates per genotype/isolate combination. Susceptibility of *B. rapa* parental lines, R500 and IMB211, to diverse *B. cinerea* isolates was digitally measured by measuring the size of the developing fungal lesion after 72 h post inoculation. The lesion size for each isolate was compared between the two genotypes using ANOVA to test the statistical significance of influence of each experimental factor, or a specific interaction between experimental factors on the lesion size. The ANOVA model was lesion = plant genotype + fungal isolate + experiment replicate + plant genotype × fungal isolate + plant genotype × experiment replicate + fungal isolate × experiment replicate + error.

For QTL mapping, three isolates that showed significantly differential virulence between the two *B. rapa* parents, Ausubel, Davis navel, and Pepper, as well as the most virulent isolate, Katie tomato, and least virulent isolate UK Razz were used to measure lesion size on each RIL in threefold replication per experiment in two independent experiments for a total of six biological replicates. Seeds were sown in replicate and plants were measured for resistance to the different isolates as described above. After planting, there were 114 RILs left for the final lesion size analysis due to the failure of 6 RILs to grow. Least square means for all lesion data was then obtained using the ANOVA as described above for the parents.

### Trypan Blue Staining

Trypan blue staining was used to visualize the growth and structure of mycelium and accompanying plant cell death caused by different *B. cinerea* isolates on leaves of both *B. rapa* parental lines. Staining of *B. rapa* leaves was performed at 12 h post-inoculation as previously described (van Wees, 2008). Briefly, the infected leaf tissues were transferred into a 50 mL plastic tube with lid and covered with 2.5 mg/mL trypan blue-lactophenol solution diluted in ethanol (96%; 1 :2 v/v). The plastic tube (lid slightly unscrewed) was heated in a boiling water bath for 1 min and the leaf tissues were left in staining solution at RT for about 12 h. Leaf tissues were destained by removing the staining solution and covering the tissues in chloral hydrate solution for 6 h and the distaining solution changed several times until the leaf tissues were clear. The cleared leaf tissues were placed into 50 mL plastic tubes with 70% glycerol. For analysis, stained leaf tissue was spread on a transparent plastic Petri dish and examined by taking high-resolution digital photos of the entire leaf tissue and each lesion

### GSL Analysis

To measure the plants response to infection, GSLs were extracted, identified and quantified using a high-throughput analytical system from all of the above leaf tissue treated with *B. cinerea* isolates, Silver nitrate or grape juice after 72 h post inoculation (Kliebenstein et al., 2001a,b,c). Briefly, GSLs were identified by comparing the retention time of HPLC peak and UV absorption spectrum with standards (Reichelt et al., 2002). For the RILs, we were only able to obtain GSL values for the control and *B. cinerea* isolate Pepper infected samples due to a technical failure during sample storage. Each GSL was analyzed using the same statistical models as for the respective lesion size analysis described above.

### QTL Analysis

To detect QTL for the lesion size and GSL content in the R500 × IMB211 RIL population, we used the least-square means for each trait for each RIL across all experiments. A high-resolution genetic map was obtained for the R500 × IMB211 RIL population from previously published resources (Devisetty et al., 2014). This was used in conjunction with the Composite interval mapping (CIM) algorithm as implemented by the cim function in the R/qtl analysis package to map QTL (Broman et al., 2003). The imputation method was selected and forward regression was used to identify three markers as covariates, with window size of 10 cM, an error of 0.0001, and 0 cM steps: cim(cross, method = “imp,” n.marcovar = 3, window = 10).

The LOD thresholds to call significant QTLs were estimated using 1000 permutation for each phenotype with a genome-wide significance level of *p* = 0.05 (Churchill and Doerge, 1994; Doerge and Churchill, 1996). Results obtained by CIM were analyzed and the define.peak function in R/eqtl analysis package was used to define the QTL with support LOD interval for each phenotypic trait (Broman et al., 2003). QTL were named with respect to their phenotypic traits and the cM position on the chromosome number. The additive effects of the loci along all chromosomes were estimated using the effectscan function in R/qtl package (Broman et al., 2003).

### Testing of QTL Interactions

To identify QTL × Isolate or QTL x QTL interactions using the detected QTL, we conducted an ANOVA using all of the RILs. In the ANOVA model, the markers that most closely associated with each QTL were used as factors. Furthermore, the different isolates as well as the untreated data were all used within the model to allow *B. cinerea* isolates and the treatments to be used as factors in the model. We tested all the QTL main effects as well as all possible pairwise interactions, including the QTL × isolate, QTL x infection or QTL × QTL interactions where appropriate.

## Results

### Variable Resistance of *B. rapa* Genotypes to Diverse *B. cinerea* Isolates

**FIGURE 1:**
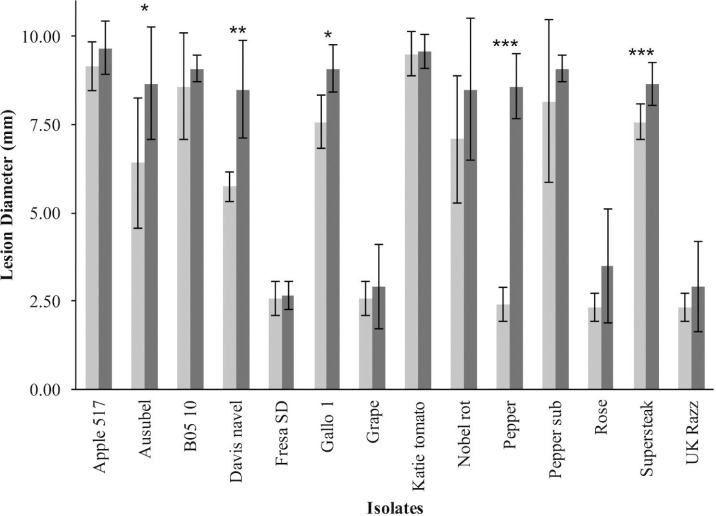
**Differential *Botrytis cinerea* virulence on the R500 and IMB211 *Brassica rapa* RIL parents.** *B. rapa*, R500 (light, left) and IMB211 (dark, right) were treated with various *B. cinerea* isolates at 72 h after inoculation and lesions quantified as described. Means of lesion size (*n* = 6) and standard error are presented. Asterisks identify isolates that have significant difference in virulence on the two *B. rapa* parents as determined using ANVOA and *post hoc* Tukey’s *t*-test to compare virulence within each isolate: ^∗^*P* < 0.05, ^∗∗^*P* < 0.01, ^∗∗∗^*P* < 0.001.

To investigate resistance to *B. cinerea*, we tested two *B. rapa* genotypes, R500 and IMB211, for resistance to 14 isolates of this necrotrophic pathogen using a previously published foliar resistance assay (**Table 1**). This detached lesion assay has been widely used to identify necrotrophic resistance genes in a number of different systems (Sharma et al., 2005; Mulema and Denby, 2012; Cowley et al., 2014; Boydom, 2015). These isolates show extensive genomic variation (Atwell et al., 2015). Leaves of *B. rapa* R500 and IMB211 were inoculated with *B. cinerea* spore suspension from each of the 14 isolates and visible expansion of necrotic lesions appeared between 12 and 24 h post inoculation (HPI), indicating outgrowth of hyphae and the initial establishment of primary lesion. Most of the lesions induced by isolates were observed to spread beyond the inoculation droplets at 24 HPI, with lesions expanding in general more rapidly on IMB211 leaves. Chlorotic zones adjacent to the developing lesion were observed for all isolates on both plant genotypes with a tendency to extend to the distal regions of leaves, plants. Quantifying lesion diameter for all infections showed that there was a statistically significant effect of the *B. rapa* and *B. cinerea* genotypes and an interaction between the plant and pathogen genotype in controlling resistance (**Table 1** and **Figure 1**). Comparison of the mean lesion diameters showed that in all instances where there was a significant effect, *B. rapa* R500 plants had smaller lesion sizes than IMB211 (**Figure 1**). In addition to the plant genotype, there were significant differences across the *B. cinerea* isolates for lesion size on the *B. rapa* genotypes ranging from the low virulence Fresa SD to higher virulence Apple 517 (**Table 1** and **Figure 1**). While most isolates showed equal virulence on the two *B. rapa* genotypes, four *B. cinerea* isolates (Ausubel, Davis Navel, Pepper and Supersteak) showed significant differences in virulence across the *B. rapa* genotypes suggesting that there are Host × pathogen genetic interactions underlying the quantitative resistance of *B. rapa* to *B. cinerea*.

**Table 1:** ANOVAs for lesion size in the *Brassica rapa* parental genotypes and recombinant inbred lines (RILs).

	Parental			RILs		
		
Sources of variation	df	SS	*P*	df	SS	*P*
Genotype	1	80	<0.001	114	2078	<0.001
Isolate	13	1137	<0.001	4	8250	<0.001
Experiment	5	8	0.256	3	1	0.556
Genotype × Isolate	13	98	<0.001	350	3005	<0.001
Genotype × Experiment	5	3	0.819	342	202	0.027
Isolate × Experiment	65	86	0.282	12	47	<0.001


*The ANOVA results for the various factors in the Parental and RIL experiments are shown with degrees of freedom (df), Type III Sums-of-Squares (SS) and estimated P-value. Genotype shows the effect of plant genetic variation while Isolate shows the effect of the pathogens genetic variation.*

### Morphological Analysis of the Interaction of *B. rapa* Genotypes with Diverse *B. cinerea* Isolates

**FIGURE 2:**
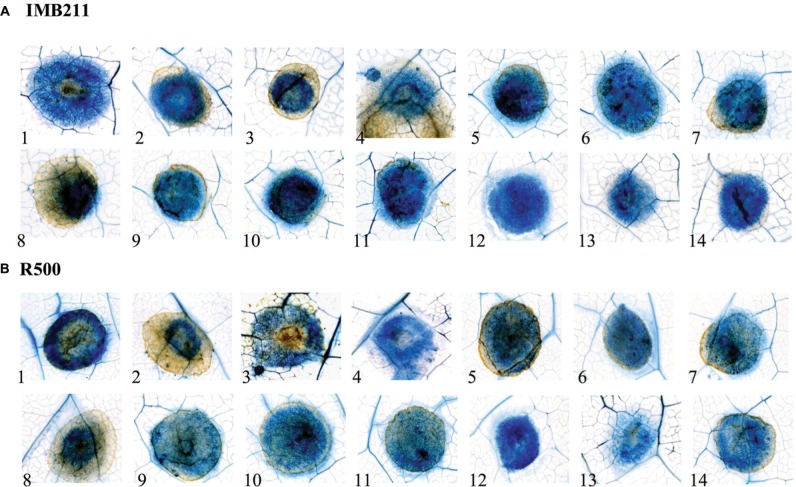
**Microscopic analysis of *B. cinerea* virulence on the R500 and IMB211 *B. rapa* RIL parents.** Lesion development on leaves of *B. rapa* parental lines **(A)** IMB211 and **(B)** R500 was assessed by Trypan blue staining at 24 h after inoculation with 14 *Botrytis* isolates. Numbers at the bottom indicate the 14 *Botrytis* isolates: 1 Apple 517, 2 Ausubel, 3 B05 10, 4 Davis navel, 5 Fresa SD, 6 Gallo 1, 7 Grape, 8 Katie tomato, 9 Nobel rot, 10 Pepper, 11 Pepper sub, 12 Rose, 13 Supersteak, 14 UK Razz.

To investigate if the quantitative variation in resistance between these *B. cinerea* isolates and *B. rapa* genotypes is apparent at the microscopic level, we stained the infected *B. rapa* leaves at 24 HPI (**Figure 2**). This allowed us to visualized plant vasculature, dead plant cells and fungal hyphae. In all cases, the fungal hyphae developed in the primary lesion, including those isolates that had extremely low virulence UK Razz, Rose or Fresa SD suggesting that the plant was preventing hyphal growth. There no consistent relationship between lesion outgrowth and hyphal production at 24 h with the strongly virulent Apple 517 and Katie Tomato showing similar hyphal distribution as the low virulent UK Razz, Rose or Fresa SD (**Figure 2**). Thus, the quantitative resistance that we are measuring in the *B. rapa*/*B. cinerea* system is not preventing hyphal germination or establishment (**Figure 2**). Instead, the quantitative resistance is altering the relative rate of outgrowth of the hyphae in each interaction. This is similar to what had previously been found in *Arabidopsis thaliana* (Rowe et al., 2010).

### QTLs for Quantitative Resistance to *B. cinerea*

**FIGURE 3:**
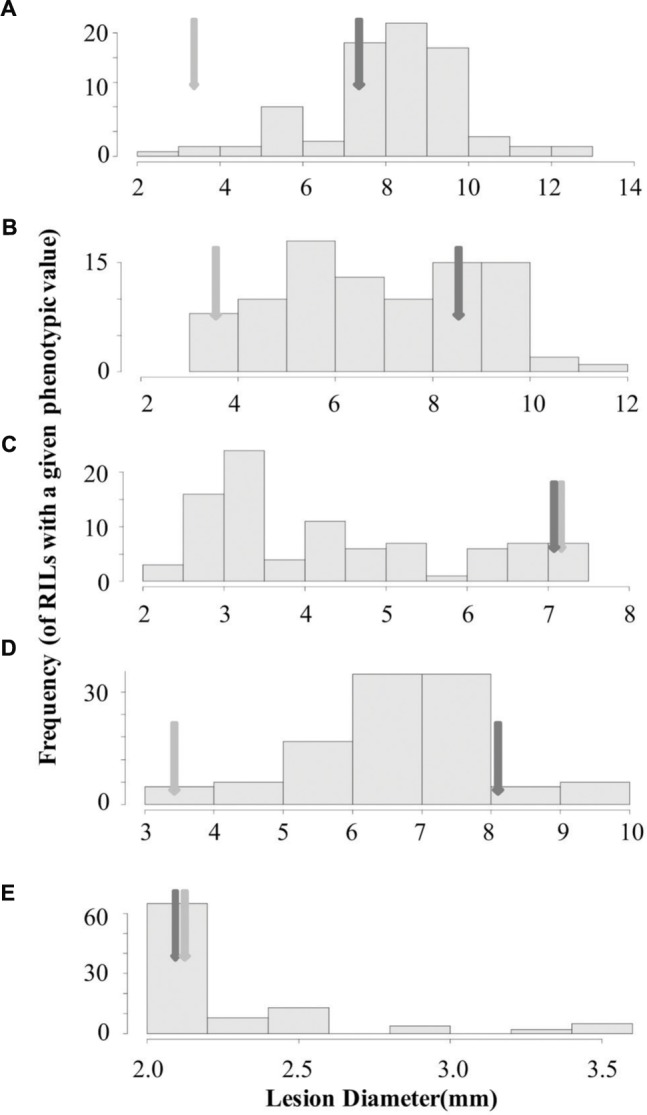
**Distribution of lesion size for five *B. cinerea* isolates on the *B. rapa* IMB211 × R500 RILs.** Distribution of mean lesion sizes (mm) on the leaves of the *B. rapa* IMB211 × R500 RILs measured at 72 h after inoculation with five *Botrytis* isolates: **(A)** Ausubel, **(B)** Davis navel, **(C)** Katie tomato, **(D)** Pepper, and **(E)** UK Razz. The dark arrow indicates the mean lesion size on IMB211 and the light arrow indicates the mean lesion size on R500.

To begin identifying the loci that may control the quantitative interaction of *B. rapa* with *B. cinerea*, we measured lesion diameter on leaves of the *B. rapa* R500 × IMB211 RIL population using five *B. cinerea* isolates (Iniguez-Luy et al., 2009). We utilized three *B. cinerea* isolates that showed different virulence on R500 and IMB211 (Ausubel, Davis Navel and Pepper) as well as one strongly virulent (Katie Tomato) and one avirulent (UK Razz) isolate that had no difference between the two parents (**Figures 1** and **3**). Lesion development induced by the five fungal isolates was significantly influenced by *B. rapa* genotypic variation (*p* < 0.001), *B. cinerea* isolate variation (*p* < 0.001) and the interaction of the two as determined using analysis of variance (ANOVA; **Table 1**). This further supports that there are genotypic dependent interactions between *B. cinerea* and *B. rapa*. The *B. rapa* RIL population displayed a range of variation for lesion size trait that was different for all five *B. cinerea* isolates (**Figure 3**). Using the Ausubel and Pepper isolates showed a distribution that skewed toward the sensitive IMB211 parent. In contrast, the Davis Navel isolate highlighted a distribution that was more evenly spread between the parental values. Interestingly, for both isolates that had no difference between the IMB211 and R500 parents, we were able to identify underlying variation in the resistance traits. For example, the Katie Tomato isolate that was equally virulent on the IMB211 and R500 parents identified a large number of RILs that transgressively segregated for increased resistance (**Figure 3**). Thus, the equal resistances of IMB211 and R500 to these two isolates are caused by opposing resistance alleles in the two *B. rapa* RIL parents.

**FIGURE 4:**
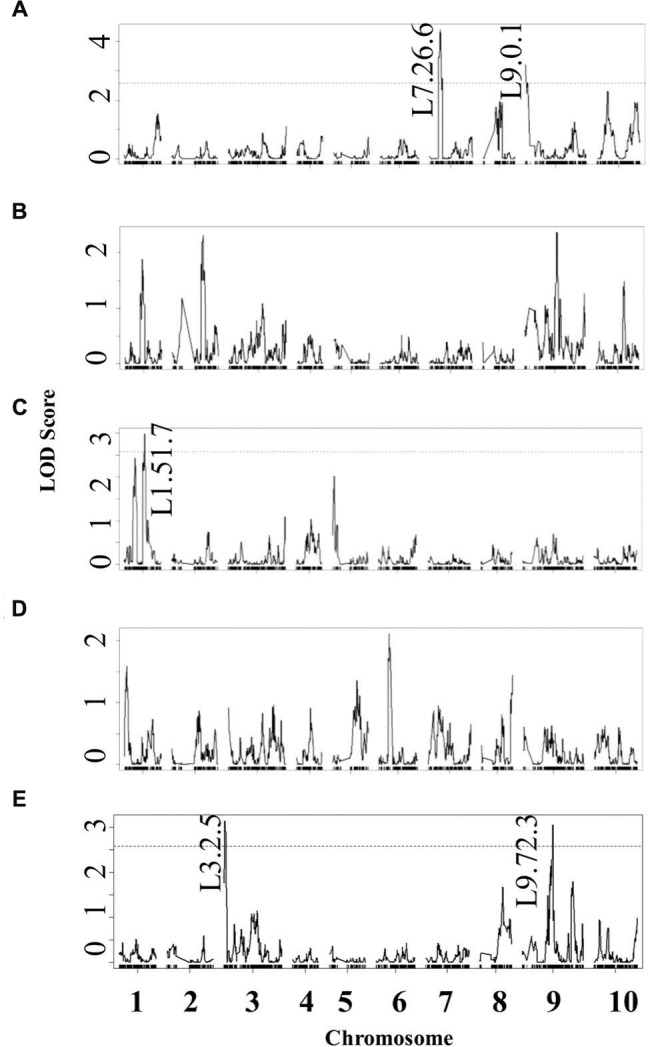
**Quantitative trait loci mapping of resistance to *B. cinerea* in *B. rapa*.** Shown are results from composite interval mapping (CIM) of the mean lesion development for all five isolates across the 115 IMB211 × R500 RILs. The QTL are labeled with their chromosome number and approximate LOD peak in cM. The LOD score is shown with the horizontal line representing the permutation obtained significance threshold. The isolates are as follows: **(A)** Ausubel, **(B)** Davis navel, **(C)** Katie tomato, **(D)** Pepper, **(E)** UK Razz.

**FIGURE 5:**
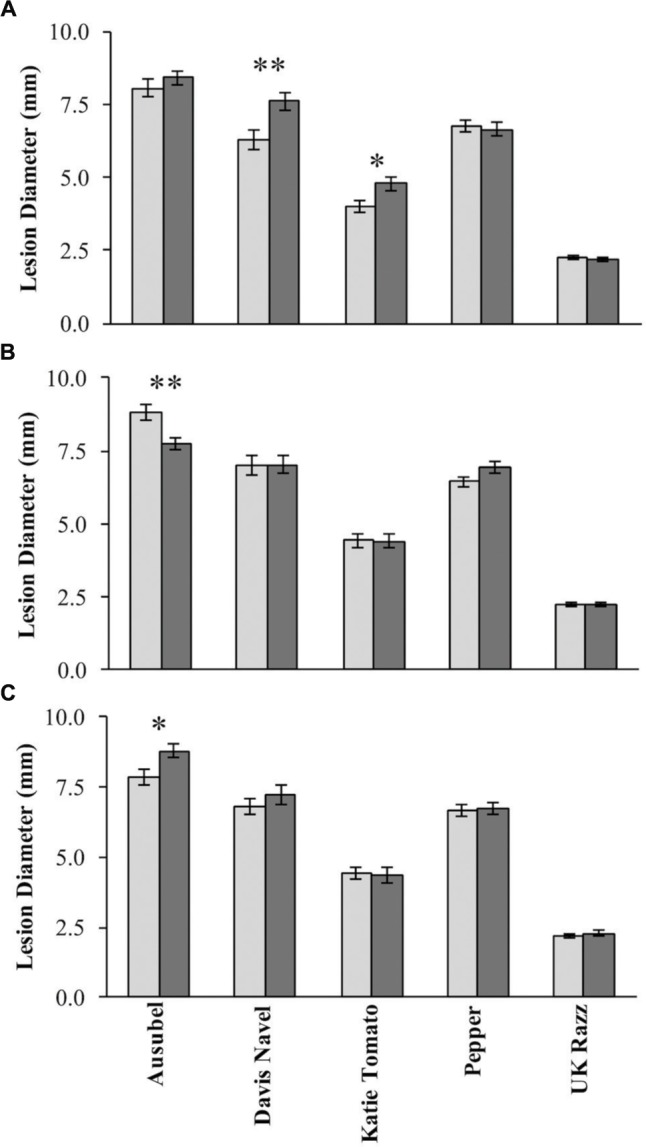
***Botrytis cinerea* isolate dependent effects of resistance QTLs.** The estimated phenotypic effect of the alleles from R500 and IMB211 at markers associated with isolate dependent QTL are shown. Error bars indicate standard error. Isolates that were significant affected by the QTL were tested by ANOVA with *post hoc* Tukey’s HSD and is indicated by asterisks above bars:^∗^*P* < 0.05, ^∗∗^*P* < 0.01. Light shows the value for the R500 allele and dark shows the value for the IMB211 allele at the given QTL. QTLs are as labeled in **Figure 4**: **(A)** QTL L1.51.7, **(B)** QTL L7.26.6, **(C)** QTL L9.0.1.

Using the lesion measurements, we mapped QTL controlling phenotypic variation in lesion size within the RILs populations derived from R500 × IMB211. This identified five QTL governing lesion size induced by *B. cinerea* isolates (**Table 3** and **Figure 4**). Two of these QTL, L7.26.6 and L9.0.1, were detected using *B.* cinerea isolate Ausubel (**Table 3** and **Figure 4**). The QTL L1.51.7 detected by *B. cinerea* isolate Katie tomato was located on chromosome I. The L3.2.5 and L9.72.3 QTL on chromosomes III and IX were detected with the *B. cinerea* isolate UK Razz isolate. Surprisingly, no QTL were detected using the *B. cinerea* isolates Davis navel and Pepper and no QTL were found consistently for all isolates. This is in contrast to the effect size plots that are similar for most isolates. This is probably due to the limited number of RILs available for this population that may generate a potentially elevated false negative rate with QTL mapping (Joseph et al., 2013, 2014). Thus, we proceeded to use a linear modeling approach to directly test if the detected QTLs were actually isolate specific. This analysis showed the QTLs were all isolate specific (**Figure 5** and **Supplementary Table 1**). Interestingly, The QTL L1.51.7 found only for the Katie Tomato isolate also significantly altered resistance to Davis Navel. Thus, we were able to identify QTL that appear to control isolate specific aspects of quantitative resistance in the interaction of *B. rapa* with *B. cinerea*.

### Identification of QTL Controlling Defense Responses

**FIGURE 6:**
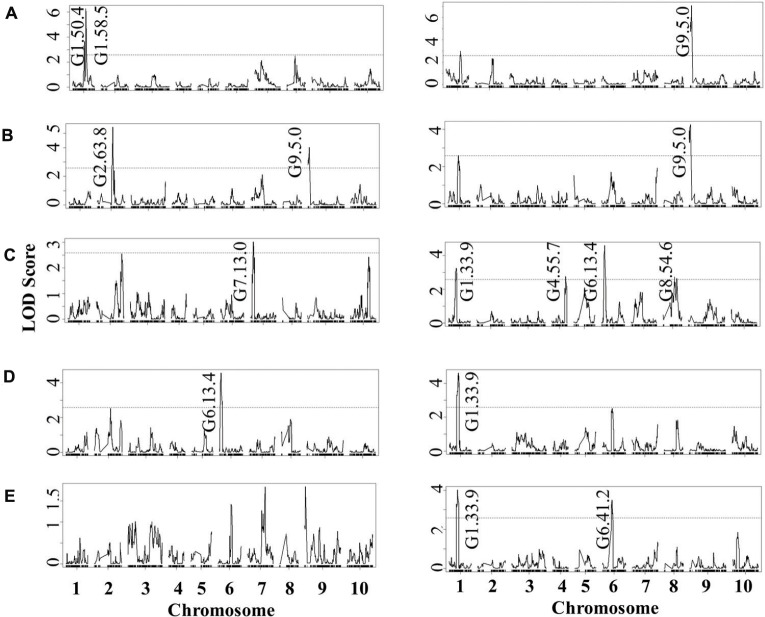
**QTL mapping of glucosinolates accumulation in *B. rapa* RILs in response to *B. cinerea.*** Shown are results from CIM of the mean glucosinolate accumulation in leaves of *B. rapa* with the absence (control, **left)** or presence (infection, **right)** of *B. cinerea* isolate Pepper across the 112 IMB211 × R500 RILs. The LOD score is shown with the horizontal line representing the permutation obtained significance threshold. Each QTL is labeled with the trait and chromosome position showing the maximum LOD score. The QTL plots for the different glucosinolates are as follows with abbreviations as given in **Table 2**: **(A)** 4MSB, **(B)** benzyl glucosinolates, **(C)** I3M, **(D)** 4MO-I3M, and **(E)** 1MO-I3M.

**FIGURE 7:**
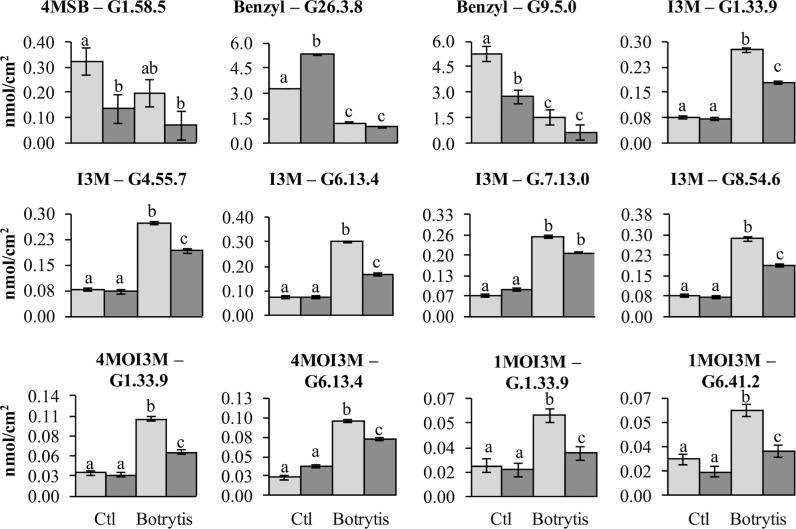
**Glucosinolates QTL that are show pathogen responsiveness.** Shown is the estimated phenotypic effect of the alleles from R500 and IMB211 at GSL accumulation QTL that are treatment dependent. Error bars indicate standard error. Significance differences between the treatment × genotype groupings were determined by ANOVA with a *post hoc* Tukey’s HSD test and are indicated with different letters. The top of each graph indicates the GSL and QTL being shown with abbreviations as listed in **Table 2**. Light shows the accumulation for RILs with the R500 allele and dark shows the value for the IMB211 allele at the given QTL.

Previous work has linked genetic variation in defense compounds to variation in biotic interactions including for *B. cinerea* (Denby et al., 2004; Rowe and Kliebenstein, 2008). Thus, we measured GSL content in all of the RILs in both control leaves and *B. cinerea* isolate Pepper infected leaves. This showed that all detected GSLs had genetic variation and were affected by the infection with the *B. cinerea* Pepper isolate (**Table 2**). There were no presence or absence polymorphisms affecting GSL abundance in this population allowing us to focus on quantitative variation controlling their relative abundance (**Table 2**; Kliebenstein et al., 2001b; Wentzell et al., 2007; Chan et al., 2010). All five GSLs identified significant QTLs with the majority of them appearing to be dependent on the presence or absence of *B. cinerea* (**Figure 6** and **Table 3**). The aliphatic and benzylic GSLs identified more QTLs under the control treatment whereas the indolic GSLs identified more QTLs under the *B. cinerea* infected tissue. This agreed with the observation that the treatments in general lead to higher indolic GSLs and lower 4MSB and Benzylic GSLs. This allowed QTL effects to be seen for indolic GSL following treatment and Benzylic and 4MSB prior to treatment (**Figure 7**). Most of the indolic QTLs were such that the IMB211 allele leads to lower pathogen induced indolic GSLs in contrast to the R500 allele (**Figure 7**). One QTL, G9.5.0, on chromosome IX shared by both aliphatic and benzylic GSLs, was also detected as affecting lesion development when the RILs were infected with the *B. cinerea* isolate Ausubel isolate. This suggested that there might be a link between these GSL and resistance to at least this *B. cinerea* isolate (**Figures 4** and **6**).

**Table 2:** ANOVAs for the accumulation of the GSLs in the *B. rapa* RILs.

		Sources of variation					
		
		Geno	Treat	Exp	Geno × Treat	Geno × Exp	Treat × Exp
GSL	df	111	1	2	111	197	2
4MSB	SS	587592	14675	4673	249079	522431	1547
	*P*	<0.001	0.009	0.329	0.331	0.052	0.691
Benzyl	SS	42514373	28489898	16266	26141049	26739529	199225
	*P*	<0.001	<0.001	0.945	0.002	0.673	0.504
I3M	SS	926625	764771	64741	863947	497570	45710
	*P*	<0.001	<0.001	<0.001	<0.001	0.764	<0.001
4MO-I3M	SS	174256	110996	3452	165917	126089	2915
	*P*	<0.001	<0.001	0.087	<0.001	0.725	0.127
1MO-I3M	SS	209265	36323	2499	152165	249352	1898
	*P*	0.042	<0.001	0.414	0.557	0.768	0.511


*The ANOVA results for the various factors that may alter GSL accumulation in the RIL experiments are shown with degrees of freedom (df), Type III Sums-of-Squares (SS) and estimated P-value. Geno shows the effect of plant genetic variation while Treat shows the effect of the presence or absence of the *Botrytis cinerea* Pepper isolate. Exp shows the effect of the experiment. 4MSB stands for 4-methylsulfinylbutyl glucosinolate, Benzyl stands for the benzylic glucosinolates (GSLs), I3M stands for Indol-3-ylmethyl glucosinolate, 4MO-I3M stands for 4-methoxy-indol-3ylmethyl glucosinolate and 1MO-I3M stands for 1-methoxy-indol-3ylmethyl glucosinolate.*

**Table 3:** *Brassica rapa* QTL governing lesion size traits and GSLs to *B. cinerea* isolates.

Trait	Chrm	QTL	Isolate	Marker	Position (cM)	LOD	Effect
Lesion size	I	L1.51.7	Katie tomato	A01_18146774	51.3–52.1	2.9	0.394
	III	L3.2.5	UK Razz	A03_356009	2.1–5.4	3.1	-0.133
	VII	L7.26.6	Ausubel	A07_9595444	21.2–29.1	4.3	-0.535
	IX	L9.0.1	Ausubel	A09_1100290	0.1–5.0	3.3	0.458
	IX	L9.72.3	UK Razz	A09_12460355	71.9–72.3	3	0.104
4MSB	I	G1.50.4	Control	A01_17216013	48.8–50.4	3.6	-0.03
	I	G1.58.5	Control	A01_20990540	55.8–58.5	6.3	-0.098
	IX	G9.5.0	Pepper	A09_140166	0.1–5.0	7.1	0.1
Benzyl	II	G2.63.8	Control	A02_12471753	61.2–65.4	5.5	1.024
	IX	G9.5.0	Pepper	A09_140166	0.1–5.0	4.3	-0.442
	IX	G9.5.0	Control	A09_140166	0.1–5.0	4	-1.24
I3M	I	G1.33.9	Pepper	A01_8502441	32.1–36.8	3.2	-0.047
	IV	G4.55.7	Pepper	A04_15348338	55.7	2.8	-0.04
	VI	G6.13.4	Pepper	A06_6167950	13.9–17.6	4.6	-0.066
	VII	G7.13.0	Control	A07_2426046	13.0–14.3	3	0.01
	VIII	G8.54.6	Pepper	A08_19941953	54.6	2.6	-0.048
4MO-I3M	I	G1.33.9	Pepper	A01_9510763	31.6–40.9	4.6	0.023
	VI	G6.13.4	Control	A06_4711632	8.1–14.8	4.6	0.007
1MO-I3M	I	G1.33.9	Pepper	A01_9277017	32.1–40.9	4	-0.014
	VI	G6.41.2	Pepper	A06_15088326	39.194–44.513	3.5	-0.015


*Shown are the identified QTLs with their Chromosome (Chrm), closest marker, genetic position, maximum LOD value and estimated effect size. 4MSB stands for 4-methylsulfinylbutyl glucosinolate, Benzyl stands for the benzylic GSLs, I3M stands for Indol-3-ylmethyl glucosinolate, 4MO-I3M stands for 4-methoxy-indol-3ylmethyl glucosinolate and 1MO-I3M stands for 1-methoxy-indol-3ylmethyl glucosinolate.*

### Epistasis Analysis

**FIGURE 8:**
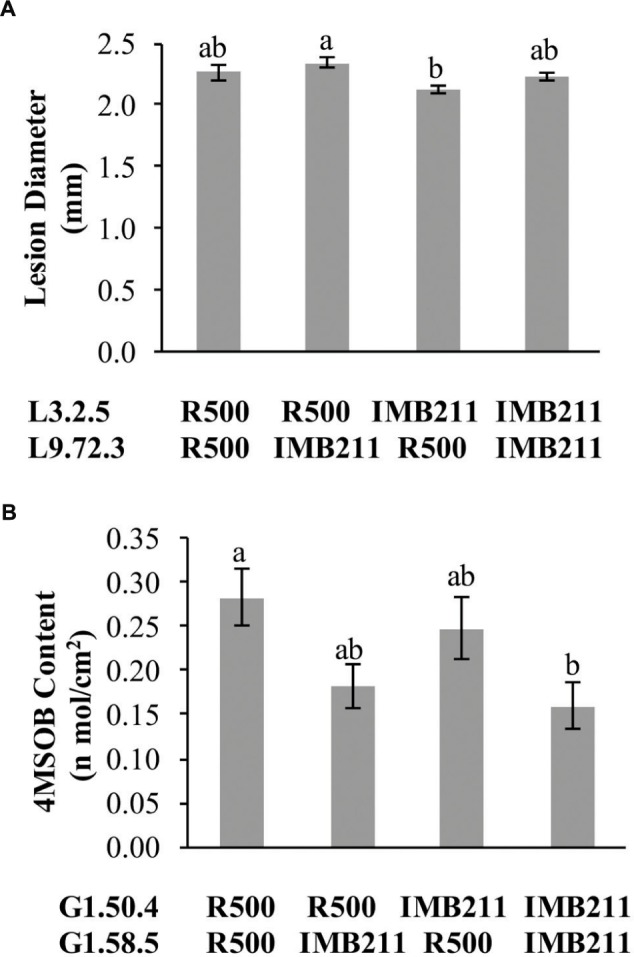
**Epistatic interactions.** The estimated phenotypic effect for loci showing epistatic interactions governing lesion size and GSL accumulation are shown. Letters in each individual graph indicate significantly different genotypes as determined by ANOVA and *post hoc* Tukey’s HSD. The *x* axis shows the allelic status of the two QTL listed to the left. **(A)** Epistatic interaction of L3.2.5 × L9.72.3 on resistance to *B. cinerea* isolate UK Razz. **(B)** Epistatic interaction of G1.50.4 × G1.58.5 on 4MSB accumulation.

Previous work on quantitative resistance to *B. cinerea* has shown that the identified QTLs are typically epistatic to each other (Finkers et al., 2007a,b, 2008; Rowe and Kliebenstein, 2008). To investigate the epistatic architecture underlying isolate-specific resistance to *B. cinerea* and chemical defense within the R500 × IMB211 population, we conducted ANOVA models for QTL controlling lesion development and individual GSL using the genetic markers closest to the QTL peak as terms in the linear model. In contrast to previous studies, we only found a single epistatic interaction with any evidence of significance in altering *Botrytis* resistance. This interaction was between the L3.2.5 and L9.72.3 QTLs that were unique to the UK Razz isolate (**Figure 8**). In comparison to *B. cinerea* resistance, more epistatic interactions were detected for GSL accumulation, one for the accumulation of aliphatic GSLs and three for the accumulation of the I3M GSL (**Figure 8**, **Supplementary Tables 2**–**6**). Interestingly, all of the epistatic interactions between GSL loci were also found to interact with the presence or absence of the pathogen suggesting that they may be linked to the regulation of the defense compounds.

## Discussion

Using 14 diverse *B. cinerea* isolates to measure quantitative resistance in two *B. rapa* lines that are the parent of a common RIL population showed that the two parents differed in their resistance to some but not all of the isolates (**Figure 1**). Further QTL mapping with a subset of these isolates showed that the genetic basis of this is likely polygenic with transgressive segregation showing that both parents can contribute resistance alleles. This was even the case when both *B. rapa* parents had identical resistance to an isolate (**Figures 3** and **4**). These loci need to be assessed in whole plant assays under field growth conditions to assess their agronomic utility. This quantitative, isolate specific and transgressive basis of quantitative resistance to *B. cinerea* is similar to what has been seen in *A. thaliana* (Denby et al., 2004; Rowe and Kliebenstein, 2008). This suggests that directly searching for QTL that provide broad spectrum resistance to *B. cinerea* within *Brassica’s* may not be the best approach to identifying successful avenues to achieve this goal. An alternative approach to utilizing these genetic loci may be to stack isolate specific loci with overlapping specificity to create a quilt of loci that can provide a protective blanket against an array of diverse isolates.

We were also able to identify QTLs that control the accumulation of the defensive glucosinolate metabolites in response to infection (**Figure 6**). The lower number of overlapped QTLs between GSLs and resistance may be due to the significant level of false negative error even in a large size of RIL population during QTL mapping (Chan et al., 2011; Joseph et al., 2013). While a few of these QTLs overlapped with one *B. cinerea* resistance QTL, GLS have frequently been linked to altered biotic interactions within the *Brassica’s* (Mithen et al., 1986, 1987; Mithen, 1992; Mithen and Magrath, 1992; Kroymann and Mitchell-Olds, 2005; Pfalz et al., 2007, 2009; Fan et al., 2011; Stotz et al., 2011; Buxdorf et al., 2013; Cargnel et al., 2014; Weis et al., 2014; Calmes et al., 2015; Kerwin et al., 2015). However, the specific mechanism by which the GSL can alter biotic interactions is not yet well understood. Some studies have provided evidence of direct toxicity to the biotic attacker that can be compensated by resistance mechanisms in the pathogen (Bednarek et al., 2009; Fan et al., 2011; Stotz et al., 2011). In contrast other studies have begun to illuminate a different possibility, specifically that the GLS alter the defense signaling pathways by which the plant responds to pathogens or jasmonic acid (Clay et al., 2009; Kerwin et al., 2011; Burow et al., 2015). Using the QTL results, it is not possible to discriminate between these two possibilities and further work will be required to test if the link between GSL accumulation and *B. cinerea* resistance in *B. rapa* is due to direct toxicity, altered defense responses or a blend of both. Partitioning between these two possibilities will be key to develop a maximally efficient effort at improving resistance to a broad array of *B. cinerea* isolates in *B. rapa*.

## Conclusion

This work begins to highlight the underlying genetic complexity of breeding for improved resistance to *B. cinerea* within *B. rapa.* The directly identified resistance loci are highly isolate specific but it may be possible to improve the breeding efficiency by breeding for an optimal defense compound blend. This, however, needs to be balanced by the fact that these same defense compounds also influence the flavor and nutritive value of the resulting *Brassica* crop. As such any effort at resistance breeding will by fact of this link also alter the quality of the resulting crop. By combining quantitative resistance loci that target overlapping ranges of *Botrytis* isolates together may be a more feasible breeding strategy to confer a broad-spectrum and durable resistance to crops against this pathogen. It is also important to note that this level of isolate specific resistance loci has significant impact on the mechanistic analysis of quantitative resistance (Broekgaarden et al., 2015). This indicates that the use of individual isolates will only provide mechanistic insight into how that individual isolate is resisted and that a broad range of pathogen genetics needs to be incorporated to assess the broader mechanistic influences on quantitative resistance. The low number of identified QTLs given the high heritability of the resistance trait suggests that there is a need to increase the available RIL population sizes to decrease the false negative error rate and to obtain a more precise picture of the genetic architecture underlying the resistance to *B. cinerea* in *B. rapa*.

## Author Contributions

S-TK, DK conceived and designed the experiments. WZ, S-TK performed the experiments. WZ, S-TK, DK analysis the data. WZ, DK wrote the paper.

## Conflict of Interest Statement

The authors declare that the research was conducted in the absence of any commercial or financial relationships that could be construed as a potential conflict of interest.
