# Supplementary material for: Isolate Dependency of Brassica rapa Resistance QTLs to Botrytis cinerea
Source: Front Plant Sci. 2016 Feb 17;7:161. doi: 10.3389/fpls.2016.00161 (PMC4756292; doi:10.3389/fpls.2016.00161)
Supplement: Supplementary file 1 [file Data_Sheet_1.DOCX]

Supplementary Material

**Isolate dependency of *Brassica rapa* resistance QTLs to *Botrytis cinerea.***

Wei Zhang*, Soon-Tae Kwon, Fang Chen, Daniel J. Kliebenstein

*** Correspondence:** Daniel J. Kliebenstein: Kliebenstein@ucdavis.edu

# Supplementary Tables

**Supplemental Table 1. ANOVA for epistatic interactions of loci governing lesion size in *B. rapa*.**

| **Source of Variation** | **QTL Name** | **df** | **SS** | **MS** | **F** | **P** |
| --- | --- | --- | --- | --- | --- | --- |
| **Isolates** |  | 4 | 2141.3 | 535.3 | 245.762 | < 0.001 *** |
| **A01_18146774** | L1.51.7 | 1 | 23.4 | 23.4 | 10.758 | 0.001 ** |
| **A03_356009** | L3.2.5 | 1 | 0 | 0 | 0.017 | 0.897 |
| **A07_9595444** | L7.26.6 | 1 | 0.9 | 0.9 | 0.427 | 0.514 |
| **A09_1100290** | L9.0.1 | 1 | 14.4 | 14.4 | 6.604 | 0.011 * |
| **A09_12460355** | L9.72.3 | 1 | 7.1 | 7.1 | 3.266 | 0.072 |
| **Isolates x A01_18146774** |  | 4 | 32.5 | 8.1 | 3.73 | 0.005 ** |
| **Isolates x A03_356009** |  | 4 | 13.5 | 3.4 | 1.547 | 0.188 |
| **Isolates x A07_9595444** |  | 4 | 25.8 | 6.5 | 2.965 | 0.020 * |
| **Isolates x A09_1100290** |  | 4 | 26.6 | 6.6 | 3.048 | 0.017 * |
| **Isolates x A09_12460355** |  | 4 | 19.9 | 5 | 2.28 | 0.06 |
| **A01_18146774 x A03_356009** |  | 1 | 0.1 | 0.1 | 0.052 | 0.82 |
| **A01_18146774 x A07_9595444** |  | 1 | 1.7 | 1.7 | 0.772 | 0.38 |
| **A01_18146774 x A09_1100290** |  | 1 | 5.4 | 5.4 | 2.46 | 0.118 |
| **A01_18146774 x A09_12460355** |  | 1 | 0.3 | 0.3 | 0.136 | 0.713 |
| **A03_356009 x A07_9595444** |  | 1 | 1.5 | 1.5 | 0.698 | 0.404 |
| **A03_356009 x A09_1100290** |  | 1 | 1.7 | 1.7 | 0.788 | 0.375 |
| **A03_356009 x A09_12460355** |  | 1 | 8.5 | 8.5 | 3.885 | 0.049 * |
| **A07_9595444 x A09_1100290** |  | 1 | 0.8 | 0.8 | 0.364 | 0.547 |
| **A07_9595444 x A09_12460355** |  | 1 | 0.2 | 0.2 | 0.071 | 0.791 |
| **A09_1100290 x A09_12460355** |  | 1 | 8.1 | 8.1 | 3.738 | 0.054 |
| **Isolates x A01_18146774 x A03_356009** |  | 4 | 10.4 | 2.6 | 1.191 | 0.315 |
| **Isolates x A01_18146774 x A07_9595444** |  | 4 | 1.2 | 0.3 | 0.139 | 0.968 |
| **Isolates x A01_18146774 x A09_1100290** |  | 4 | 10.1 | 2.5 | 1.155 | 0.33 |
| **Isolates x A01_18146774 x A09_12460355** |  | 4 | 9.2 | 2.3 | 1.06 | 0.376 |
| **Isolates x A03_356009 x A07_9595444** |  | 4 | 12.9 | 3.2 | 1.477 | 0.209 |
| **Isolates x A03_356009 x A09_1100290** |  | 4 | 4.8 | 1.2 | 0.555 | 0.696 |
| **Isolates x A03_356009 x A09_12460355** |  | 4 | 16.6 | 4.1 | 1.901 | 0.11 |
| **Isolates x A07_9595444 x A09_1100290** |  | 4 | 6 | 1.5 | 0.684 | 0.604 |
| **Isolates x A07_9595444 x A09_12460355** |  | 4 | 1.4 | 0.4 | 0.161 | 0.958 |
| **Isolates x A09_1100290 x A09_12460355** |  | 4 | 6.3 | 1.6 | 0.718 | 0.58 |
| **A01_18146774 x A03_356009 x A07_9595444** |  | 1 | 4.2 | 4.2 | 1.949 | 0.164 |
| **A01_18146774 x A03_356009 x A09_1100290** |  | 1 | 1.7 | 1.7 | 0.763 | 0.383 |
| **A01_18146774 x A03_356009 x A09_12460355** |  | 1 | 0.2 | 0.2 | 0.069 | 0.793 |
| **A03_356009 x A07_9595444 x A09_1100290** |  | 1 | 7.5 | 7.5 | 3.422 | 0.065 |
| **A03_356009 x A07_9595444 x A09_12460355** |  | 1 | 2.8 | 2.8 | 1.265 | 0.262 |
| **A03_356009 x A09_1100290 x A09_12460355** |  | 1 | 0.2 | 0.2 | 0.098 | 0.755 |
| **A07_9595444 x A09_1100290 x A09_12460355** |  | 1 | 0.7 | 0.7 | 0.302 | 0.583 |
| **Residuals** |  | 366 | 797.2 | 2.2 |  |  |

**Supplemental Table 2. ANOVA for epistatic interactions of loci governing glucosinolate (4-methyl sulfinyl butyl) contents in *B.rapa*.**

| **Source of Variation** | **QTL Name** | **df** | **SS** | **MS** | **F** | **P** |
| --- | --- | --- | --- | --- | --- | --- |
| **Treatments** |  | 1 | 0.45 | 0.45 | 9.685 | 0.002 ** |
| **A01_17216013** | G1.50.4 | 1 | 0.315 | 0.315 | 6.786 | 0.01 ** |
| **A01_20990540** | G1.58.5 | 1 | 1.723 | 1.723 | 37.119 | < 0.001 *** |
| **A09_140166** | G9.5.0 | 1 | 1.404 | 1.404 | 30.243 | < 0.001 *** |
| **Treatments x A01_17216013** |  | 1 | 0.023 | 0.023 | 0.5 | 0.48 |
| **Treatments x A01_20990540** |  | 1 | 0.4 | 0.4 | 8.611 | 0.004 ** |
| **Treatments x A09_140166** |  | 1 | 0.06 | 0.06 | 1.292 | 0.257 |
| **A01_17216013 x A01_20990540** |  | 1 | 0.941 | 0.941 | 20.268 | < 0.001 *** |
| **A01_17216013 x A09_140166** |  | 1 | 0.104 | 0.104 | 2.234 | 0.137 |
| **A01_20990540 x A09_140166** |  | 1 | 0.161 | 0.161 | 3.471 | 0.0639 |
| **Treatments x A01_17216013 x A01_20990540** |  | 1 | 0.996 | 0.995 | 21.445 | < 0.001 *** |
| **Treatments x A01_17216013 x A09_140166** |  | 1 | 0 | 0.001 | 0.01 | 0.92 |
| **Residuals** |  | 203 | 9.423 | 0.046 |  |  |

**Supplemental Table 3. ANOVA for epistatic interactions of loci governing benzyl glucosinolates contents in *B.rapa*.**

| **Source of Variation** | **QTL Name** | **df** | **SS** | **MS** | **F** | **P** |
| --- | --- | --- | --- | --- | --- | --- |
| **Treatments** |  | 1 | 478.4 | 478.4 | 118.369 | < 0.001 *** |
| **A02_12471753** | G2.63.8 | 2 | 52.5 | 26.3 | 6.499 | 0.002 ** |
| **A09_140166** | G9.5.0 | 1 | 146.1 | 146.1 | 36.145 | < 0.001 *** |
| **Treatments x A02_12471753** |  | 2 | 71.5 | 35.7 | 8.843 | < 0.001 *** |
| **Treatments x A09_140166** |  | 1 | 31.2 | 31.2 | 7.712 | 0.006 ** |
| **A02_12471753 x A09_140166** |  | 2 | 3 | 1.5 | 0.373 | 0.689 |
| **Treatments x A02_12471753 x A09_140166** |  | 2 | 19.8 | 9.9 | 2.451 | 0.089 |
| **Residuals** |  | 204 | 824.4 | 4 |  |  |

**Supplemental Table 4. ANOVA for epistatic interactions of loci governing I3M GSL accumulation in *B. rapa*.**

| **Source of Variation** | **QTL Name** | **df** | **SS** | **MS** | **F** | **P** |
| --- | --- | --- | --- | --- | --- | --- |
| **Treatments** |  | 1 | 1.3388 | 1.3388 | 163.526 | < 0.001 *** |
| **A01_8502441** | G1.33.9 | 1 | 0.1272 | 0.1272 | 15.533 | < 0.001 *** |
| **A04_15348338** | G4.55.7 | 1 | 0.1453 | 0.1453 | 17.752 | < 0.001 *** |
| **A06_6167950** | G6.13.4 | 1 | 0.2679 | 0.2679 | 32.726 | < 0.001 *** |
| **A07_2426046** | G7.13.0 | 1 | 0.0052 | 0.0052 | 0.636 | 0.426 |
| **A08_19941953** | G8.54.6 | 2 | 0.1805 | 0.0902 | 11.022 | < 0.001 *** |
| **Treatments x A01_8502441** |  | 1 | 0.1106 | 0.1106 | 13.509 | < 0.001 *** |
| **Treatments x A04_15348338** |  | 1 | 0.1147 | 0.1147 | 14.005 | < 0.001 *** |
| **Treatments x A06_6167950** |  | 1 | 0.2456 | 0.2456 | 29.994 | < 0.001 *** |
| **Treatments x A07_2426046** |  | 1 | 0.045 | 0.045 | 5.491 | 0.020 * |
| **Treatments x A08_19941953** |  | 2 | 0.1404 | 0.0702 | 8.575 | < 0.001 *** |
| **A01_8502441 x A04_15348338** |  | 1 | 0.0278 | 0.0278 | 3.398 | 0.067 |
| **A01_8502441 x A06_6167950** |  | 1 | 0.0015 | 0.0015 | 0.189 | 0.664 |
| **A01_8502441 x A07_2426046** |  | 1 | 0.0055 | 0.0055 | 0.669 | 0.414 |
| **A01_8502441 x A08_19941953** |  | 2 | 0.0676 | 0.0338 | 4.126 | 0.018 * |
| **A04_15348338 x A06_6167950** |  | 1 | 0.0393 | 0.0393 | 4.795 | 0.030 * |
| **A04_15348338 x A07_2426046** |  | 1 | 0.0052 | 0.0052 | 0.64 | 0.425 |
| **A04_15348338 x A08_19941953** |  | 1 | 0.0375 | 0.0375 | 4.581 | 0.0338 * |
| **A06_6167950 x A07_2426046** |  | 1 | 0.0285 | 0.0285 | 3.481 | 0.064 |
| **A06_6167950 x A08_19941953** |  | 1 | 0.0504 | 0.0504 | 6.159 | 0.014 * |
| **A07_2426046 x A08_19941953** |  | 1 | 0 | 0 | 0.004 | 0.948 |
| **Treatments x A01_8502441 x A04_15348338** |  | 1 | 0.0288 | 0.0288 | 3.523 | 0.062 |
| **Treatments x A01_8502441 x A06_6167950** |  | 1 | 0.0111 | 0.0111 | 1.352 | 0.247 |
| **Treatments x A01_8502441 x A07_2426046** |  | 1 | 0.0023 | 0.0023 | 0.275 | 0.6 |
| **Treatments x A01_8502441 x A08_19941953** |  | 2 | 0.0521 | 0.0261 | 3.182 | 0.044 * |
| **Treatments x A04_15348338 x A06_6167950** |  | 1 | 0.0633 | 0.0633 | 7.731 | 0.006 ** |
| **Treatments x A04_15348338 x A07_2426046** |  | 1 | 0.0069 | 0.0069 | 0.844 | 0.359 |
| **Treatments x A04_15348338 x A08_19941953** |  | 1 | 0.0378 | 0.0378 | 4.612 | 0.033 * |
| **Treatments x A06_6167950 x A07_2426046** |  | 1 | 0.0035 | 0.0035 | 0.43 | 0.513 |
| **Treatments x A06_6167950 x A08_19941953** |  | 1 | 0.0428 | 0.0428 | 5.227 | 0.023 * |
| **Treatments x A07_2426046 x A08_19941953** |  | 1 | 0 | 0 | 0.001 | 0.977 |
| **A01_8502441 x A04_15348338 x A06_6167950** |  | 1 | 0.0007 | 0.0007 | 0.082 | 0.775 |
| **A01_8502441 x A04_15348338 x A07_2426046** |  | 1 | 0.0006 | 0.0006 | 0.074 | 0.785 |
| **A01_8502441 x A04_15348338 x A08_19941953** |  | 1 | 0.0119 | 0.0119 | 1.455 | 0.229 |
| **A01_8502441 x A06_6167950 x A07_2426046** |  | 1 | 0.0002 | 0.0002 | 0.026 | 0.872 |
| **A01_8502441 x A06_6167950 x A08_19941953** |  | 1 | 0.0001 | 0.0001 | 0.011 | 0.918 |
| **A01_8502441 x A07_2426046 x A08_19941953** |  | 1 | 0.03 | 0.03 | 3.658 | 0.057 |
| **A04_15348338 x A06_6167950 x A07_2426046** |  | 1 | 0.0021 | 0.0021 | 0.252 | 0.616 |
| **A04_15348338 x A06_6167950 x A08_19941953** |  | 1 | 0.0326 | 0.0326 | 3.979 | 0.048 * |
| **A04_15348338 x A07_2426046 x A08_19941953** |  | 1 | 0.0023 | 0.0023 | 0.275 | 0.601 |
| **A06_6167950 x A07_2426046 x A08_19941953** |  | 1 | 0.0012 | 0.0012 | 0.141 | 0.707 |
| **Residuals** |  | 170 | 1.3918 | 0.0082 |  |  |

**Supplemental Table 5. ANOVA for epistatic interactions of loci governing 4MO-I3M GSL accumulation in *B. rapa*.**

| **Source of Variation** | **QTL Name** | **df** | **SS** | **MS** | **F** | **P** |
| --- | --- | --- | --- | --- | --- | --- |
| **Treatments** |  | 1 | 0.1472 | 0.1472 | 77.021 | < 0.001 *** |
| **A01_9510763** | G1.33.9 | 1 | 0.0315 | 0.0315 | 16.464 | < 0.001 *** |
| **A06_4711632** | G6.13.4 | 1 | 0.0015 | 0.0015 | 0.8 | 0.372 |
| **Treatments x A01_9510763** |  | 1 | 0.026 | 0.026 | 13.628 | < 0.001 *** |
| **Treatments x A06_4711632** |  | 1 | 0.0192 | 0.0192 | 10.023 | 0.002 ** |
| **A01_9510763 x A06_4711632** |  | 1 | 0.0055 | 0.0055 | 2.852 | 0.093 |
| **Treatments x A01_9510763 x A06_4711632** |  | 1 | 0.0007 | 0.0007 | 0.374 | 0.542 |
| **Residuals** |  | 208 | 0.3975 | 0.0019 |  |  |

**Supplemental Table 6. ANOVA for epistatic interactions of loci governing 1MO-I3M GSL accumulation in *B. rapa*.**

| **Sources of Variation** | **QTL name** | **df** | **SS** | **MS** | **F** | **P** |
| --- | --- | --- | --- | --- | --- | --- |
| **Treatments** |  | 1 | 0.03001 | 0.030015 | 22.943 | < 0.001 *** |
| **A01_9277017** | G1.33.9 | 1 | 0.01221 | 0.012212 | 9.334 | 0.00254 ** |
| **A06_15088326** | G6.41.2 | 1 | 0.02273 | 0.022731 | 17.375 | < 0.001 *** |
| **Treatments x A01_9277017** |  | 1 | 0.00779 | 0.007791 | 5.956 | 0.01551 * |
| **Treatments x A06_15088326** |  | 1 | 0.00653 | 0.006532 | 4.993 | 0.02651 * |
| **A01_9277017 x A06_15088326** |  | 1 | 0.00217 | 0.002169 | 1.658 | 0.19934 |
| **Treatments x A01_9277017 x A06_15088326** |  | 1 | 0.00112 | 0.001124 | 0.859 | 0.35498 |
| **Residuals** |  | 208 | 0.27211 | 0.001308 |  |  |
